# Performance Comparison of a Neuro-Symbolic Large Language Model System Versus Human Experts in Acute Cholecystitis Management

**DOI:** 10.3390/jcm15051730

**Published:** 2026-02-25

**Authors:** Evren Ekingen, Mete Ucdal

**Affiliations:** 1 Department of Emergency Medicine, Etimesgut Sehit Sait Erturk State Hospital, Ankara 06790, Turkey; evren23@gmail.com; 2 Department of Internal Medicine, Etimesgut Şehit Sait Erturk State Hospital, Ankara 06790, Turkey

**Keywords:** acute cholecystitis, artificial intelligence, large language models, neuro-symbolic AI, Tokyo Guidelines 2018, clinical decision support, Gallbladder diseases

## Abstract

**Background/Objectives:** Large language models (LLMs) have shown promising results in medical decision support; however, their effectiveness in managing acute cholecystitis and other gallbladder diseases remains insufficiently examined. This study evaluated the performance of a neuro-symbolic LLM system that integrates multiple AI agents with neural–symbolic reasoning for acute cholecystitis management and compared its diagnostic accuracy with that of human expert physicians across three clinical specialties. **Methods:** This multi-center cross-sectional study included 30 case-based questions covering acute cholecystitis and gallbladder diseases, stratified across eight predefined disease categories: acute calculous cholecystitis (n = 6), acute acalculous cholecystitis (n = 2), complicated cholecystitis including gangrenous, emphysematous, and perforated variants (n = 5), chronic cholecystitis and biliary colic (n = 4), gallbladder polyps and adenomyomatosis (n = 3), Mirizzi syndrome (n = 2), gallbladder carcinoma (n = 4), and post-cholecystectomy complications (n = 4). Questions were categorized into diagnosis (n = 10), treatment (n = 10), and complications/prognosis (n = 10). Gold standard answers were established through consensus by an expert panel consisting of two senior general surgery expert clinicians and one senior emergency medicine expert clinician, each with more than 20 years of clinical experience, utilizing the Tokyo Guidelines 2018 (TG18) as the reference standard for diagnostic criteria, severity grading, and management recommendations. The expert panel achieved unanimous consensus on all 30 gold standard answers. All responses were cross-referenced against the primary TG18 publications to ensure guideline-based rather than solely opinion-based reference standards. This consensus-based, guideline-anchored approach is consistent with established methodologies for gold standard establishment in AI diagnostic accuracy studies. Performance of a neuro-symbolic LLM system orchestrated via LangGraph v1.0 was compared against 10 general surgery specialists, 10 emergency medicine physicians, and 10 gastroenterology specialists from four tertiary centers in Turkey. The neuro-symbolic system incorporated the Tokyo Guidelines 2018 (TG18) as its symbolic knowledge base for diagnostic criteria, severity grading, and management algorithms. **Results:** The neuro-symbolic system attained the highest overall accuracy rate of 96.7% (29/30), markedly surpassing the performance of general surgery specialists (average 82.3% ± 6.8%), emergency medicine physicians (average 71.0% ± 8.2%), and gastroenterology specialists (average 78.7% ± 7.4%). Furthermore, the neuro-symbolic system exhibited superior performance across all clinical categories. Among human participants, general surgeons showed the highest accuracy in treatment decisions (88.0%), while gastroenterologists excelled in diagnostic questions (82.0%). Emergency medicine physicians showed comparable performance to other specialties in acute presentation scenarios. ROC analysis revealed excellent discrimination for the neuro-symbolic system (AUC = 0.983) compared to general surgery (AUC = 0.856), gastroenterology (AUC = 0.821), and emergency medicine (AUC = 0.764). **Conclusions:** The neuro-symbolic LLM system exhibited superior performance in standardized guideline-concordant case-based assessment of acute cholecystitis management compared to all human expert groups, reflecting its consistent application of encoded guideline criteria. These findings support its potential role as a clinical decision-support tool that augments, rather than replaces, physician expertise. The system’s consistent application of standardized guidelines indicates its potential utility as a clinical decision support tool, particularly in settings where specialist expertise is limited. However, these results should be interpreted within the constraints of a structured case-based evaluation and do not imply global clinical superiority over human experts.

## 1. Introduction

Acute cholecystitis is among the most prevalent surgical emergencies globally, affecting approximately 10–15% of the adult population with gallstones and accounting for 3–10% of all patients presenting with abdominal pain at emergency departments [1]. The Tokyo Guidelines 2018 (TG18) offer an internationally acknowledged, evidence-based framework for the diagnosis, severity assessment, and management of acute cholecystitis [2]. Despite the existence of these standardized guidelines, considerable variability remains in clinical practice, with adherence rates reported to range from 45% to 78% across diverse healthcare settings [3]. This inconsistency may be due to the complexity involved in integrating multiple clinical parameters, the time limitations in acute care environments, and the cognitive demands associated with the application of algorithmic decision-making protocols.

Large language models (LLMs) have emerged as promising tools to support healthcare delivery, from automating tasks to aiding clinical decision-making [4]. Recent studies have demonstrated their potential in clinical decision support, diagnostic reasoning, and treatment recommendations across various medical specialties [5,6]. However, conventional LLMs exhibit significant limitations, including factual hallucinations, inconsistent guideline application, and inability to reliably execute multi-step diagnostic algorithms [7]. A recent multi-national study demonstrated that LLMs can elaborate on fabricated clinical details in 50–82% of cases, highlighting their vulnerability to generating misleading medical content [8]. These limitations are particularly consequential in acute cholecystitis management, where misdiagnosis or inappropriate severity grading may result in delayed intervention or unnecessary surgery.

To address these limitations, neuro-symbolic artificial intelligence (AI) has emerged as a promising paradigm that amalgamates the pattern recognition capabilities of neural networks with the logical reasoning and explicit knowledge representation characteristic of symbolic AI [9]. Unlike approaches solely based on neural networks, neuro-symbolic systems are capable of parsing clinical vignettes utilizing neural components, extracting pertinent features, and mapping these onto symbolic knowledge graphs that encode medical guidelines [10]. This integration facilitates explicit and auditable clinical reasoning [11]. Recent research has substantiated the feasibility of such architectures for medical diagnosis prediction, demonstrating enhancements in accuracy, reliability, and explainability relative to conventional large language models [12].

This multi-center, cross-sectional study assessed the efficacy of a neuro-symbolic large language model (NS-LLM) system in the management of acute cholecystitis, comparing its performance to that of renowned expert physicians from three distinct clinical specialties: general surgery, emergency medicine, and gastroenterology. The neuro-symbolic system employs a multi-agent framework orchestrated via LangGraph to coordinate multiple base LLMs and incorporates a structured symbolic knowledge base that encodes the complete TG18 diagnostic criteria, severity grading system, and management algorithms. Performance was evaluated using 30 validated case-based questions encompassing diagnosis, treatment, and complications of acute cholecystitis and related gallbladder diseases. It was hypothesized that the neuro-symbolic system would attain superior performance in standardized guideline-concordant assessments by overcoming the fundamental limitations inherent in purely neural methodologies through the explicit encoding of clinical guidelines.

## 2. Methods

### 2.1. Study Design and Setting

This multi-center cross-sectional diagnostic accuracy study was conducted between October 2025 and January 2026 at four tertiary healthcare centers in Turkey: Ankara Bilkent City Hospital, Ankara Etlik City Hospital, Elazığ Fethi Sekin City Hospital, and Ankara Etimesgut Şehit Sait Ertürk State Hospital. The study was designed in accordance with the Standards for Reporting Diagnostic Accuracy Studies (STARD) guidelines and adhered to the Strengthening the Reporting of Observational Studies in Epidemiology (STROBE) checklist for cross-sectional studies. Institutional ethics committee approval was obtained from Ankara Provincial Health Directorate Ethics Committee (Protocol No: E-2025-847, Date: 15 October 2025). The study was conducted in compliance with the Declaration of Helsinki. Written informed consent was obtained from all 30 participating physicians prior to enrollment. No patients were directly enrolled in this study; all clinical vignettes were hypothetical case-based scenarios developed for assessment purposes.

### 2.2. Participants

#### 2.2.1. Human Expert Selection

Thirty physicians were recruited from four tertiary care centers in Turkey, stratified into three specialty groups of ten participants each. The general surgery group included specialists from Ankara Bilkent City Hospital (n = 3), Ankara Etlik City Hospital (n = 3), Elazığ Fethi Sekin City Hospital (n = 2), and Etimesgut Şehit Sait Ertürk State Hospital (n = 2). The emergency medicine and gastroenterology groups followed identical institutional distributions.

#### 2.2.2. Inclusion and Exclusion Criteria

General surgery specialists were required to hold board certification in general surgery with a minimum of five years of clinical experience following residency completion. Participants were expected to maintain active clinical practice involving hepatobiliary surgery and perform laparoscopic cholecystectomy regularly with a minimum of 50 cases annually. Current affiliation with a tertiary care center was mandatory.

Emergency medicine specialists were required to hold board certification in emergency medicine with a minimum of five years of clinical experience. Participants were expected to have regular involvement in the management of acute biliary emergencies and employment at a tertiary care center with 24 h surgical capability.

Gastroenterology specialists were required to hold board certification in gastroenterology or hepatology with a minimum of five years of clinical experience following fellowship completion. Participants were expected to maintain active clinical practice involving hepatobiliary disorders and current affiliation with a tertiary care center performing endoscopic retrograde cholangiopancreatography.

**Figure 1:**
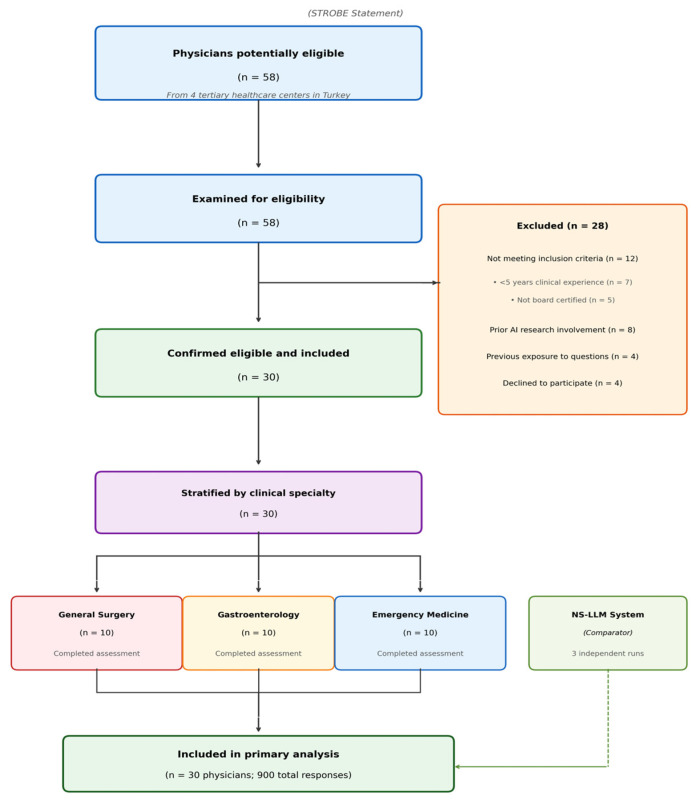
Flow Diagram of Study Participants (STROBE Statement). Between October 2025 and January 2026, 58 physicians from four tertiary healthcare centers in Turkey were assessed for eligibility. Following application of inclusion and exclusion criteria, 28 physicians were excluded: 12 did not meet inclusion criteria (7 with <5 years clinical experience, 5 without board certification), 8 had prior artificial intelligence research involvement, 4 had previous exposure to study questions, and 4 declined to participate. The final study population comprised 30 physicians stratified into three specialty groups (general surgery, n = 10; gastroenterology, n = 10; emergency medicine, n = 10). The neuro-symbolic large language model (NS-LLM) system served as the comparator, with performance assessed across three independent evaluation runs. All 30 human participants completed the 30-question assessment instrument, generating 900 total responses for primary analysis.

Exclusion criteria applied uniformly to all participant groups. Physicians with prior involvement in artificial intelligence-related clinical research were excluded to minimize potential bias. Participants with previous exposure to the study questions were excluded to ensure assessment validity. Individuals with conflicts of interest involving artificial intelligence technology companies were also excluded. The exclusion of physicians with prior involvement in artificial intelligence-related clinical research was implemented to minimize performance bias, as familiarity with AI reasoning patterns could influence responses to structured clinical vignettes in ways unrepresentative of routine clinical practice. We acknowledge that this criterion may introduce selection bias by excluding technologically engaged physicians. Physicians meeting the predefined eligibility criteria were identified through purposive sampling at participating institutions and invited to participate. Recruitment was conducted by the principal investigators at each center through direct invitation, following verification of eligibility criteria including board certification status, years of clinical experience, and annual case volume. This purposive sampling strategy was chosen to ensure that all participants met the minimum expertise thresholds necessary for meaningful comparison with the AI system. The complete study design and participant flow are illustrated in Figure 1.

### 2.3. Test Instrument

The evaluation instrument consisted of 30 case-based multiple-choice questions focusing on acute cholecystitis and gallbladder diseases. Each question presented a detailed clinical vignette including patient demographics, presenting symptoms, physical examination findings, laboratory results, and imaging reports. Questions were stratified by clinical domain: diagnosis (n = 10), treatment (n = 10), and complications/prognosis (n = 10).

Clinical scenarios represented the full spectrum of gallbladder pathology: acute calculous cholecystitis (n = 6), acute acalculous cholecystitis (n = 2), complicated cholecystitis including gangrenous, emphysematous, and perforated variants (n = 5), chronic cholecystitis and biliary colic (n = 4), gallbladder polyps and adenomyomatosis (n = 3), Mirizzi syndrome (n = 2), gallbladder carcinoma (n = 4), and post-cholecystectomy complications (n = 4). The scope of clinical scenarios was intentionally broad to reflect the real-world diagnostic challenges encountered in hepatobiliary emergencies, where differential diagnosis extends beyond isolated acute cholecystitis to encompass the full spectrum of gallbladder pathology that clinicians must navigate in clinical practice.

The case-based questions were developed by a gastroenterology subspecialist with expertise in hepatobiliary disorders. Gold standard answers were established through consensus by an expert panel consisting of two senior general surgery expert clinicians and one senior emergency medicine expert clinician, each with more than 20 years of clinical experience. The panel utilized the Tokyo Guidelines 2018 (TG18) as the reference standard for diagnostic criteria, severity grading, and management recommendations. The complete dataset including all 30 clinical vignettes, response distributions, and individual participant performance data is provided in Supplementary Table S1.

### 2.4. Neuro-Symbolic Large Language Model System Architecture

#### 2.4.1. Overview

The NS-LLM system was developed as a multi-agent orchestration framework based on the LangGraph architecture, an open-source library designed for constructing stateful, multi-agent applications utilizing large language models. The system integrates neural language processing capabilities with symbolic knowledge representation to enable explicit, auditable clinical reasoning.

#### 2.4.2. Multi-Agent Architecture

The system employs a parallel agent deployment strategy utilizing two large language models: Gemini 3.0 Flash (Google DeepMind, 2025) and GPT-5.2 (OpenAI, 2025). The selection of these two models was based on their status as the most recent and highest-performing general-purpose LLMs at the time of study design, and the deliberate pairing of architecturally distinct models from different developers to maximize reasoning diversity within the multi-agent framework. While earlier models such as Med-PaLM, LLaMA, and Claude have demonstrated notable performance in medical benchmarks, the neuro-symbolic architecture employed in this study was designed to augment base LLM capabilities through symbolic knowledge integration, making the comparative evaluation of different LLM backbones a relevant but distinct research question for future investigation. Each agent operates as an independent reasoning entity that receives identical clinical vignettes as input. The agents process clinical information through their respective neural architectures and generate candidate answers accompanied by calibrated confidence scores ranging from 0 to 1.

This dual-agent approach serves two purposes. First, it provides redundancy that reduces single-point-of-failure errors inherent in individual LLM outputs. Second, it enables cross-validation of reasoning pathways, as agreement between architecturally distinct models increases confidence in the generated response. The agents communicate through a shared state object maintained by LangGraph, which tracks intermediate reasoning steps, extracted clinical features, and provisional diagnoses throughout the inference pipeline.

#### 2.4.3. Symbolic Knowledge Base

The symbolic reasoning component incorporates a structured knowledge graph that encodes the TG18 clinical criteria for acute cholecystitis. Clinical findings, laboratory thresholds, severity grades, and management decisions are represented as interconnected nodes, while directed edges represent diagnostic implications, severity escalation pathways, and treatment algorithms. This graph structure enables efficient traversal of clinical decision pathways and supports rule-based inference that mirrors established diagnostic reasoning patterns. The knowledge base comprises four primary modules.

#### 2.4.4. Diagnostic Criteria Module

This module encodes the TG18/TG13 three-domain requirement system. Domain A encompasses local signs of inflammation including Murphy’s sign and right upper quadrant mass, pain, or tenderness. Domain B encompasses systemic signs of inflammation including fever greater than 38 °C, elevated C-reactive protein greater than or equal to 3 mg/dL, and elevated white blood cell count greater than 10,000/μL. Domain C encompasses imaging findings characteristic of acute cholecystitis including gallbladder wall thickening greater than or equal to 4 mm, enlarged gallbladder, pericholecystic fluid, incarcerated gallstone, sonographic Murphy’s sign, and debris echo. The diagnostic classification rules encode that suspected acute cholecystitis requires at least one criterion from Domain A and at least one criterion from Domain B, while definite acute cholecystitis additionally requires positive Domain C findings.

#### 2.4.5. Severity Grading Module

This module implements the TG18/TG13 three-tier classification system. Grade III (severe) acute cholecystitis is defined by dysfunction of any organ system including cardiovascular dysfunction requiring dopamine greater than or equal to 5 μg/kg/min or any dose of norepinephrine, neurological dysfunction manifesting as decreased level of consciousness, respiratory dysfunction with PaO_2_/FiO_2_ ratio less than 300, renal dysfunction with oliguria or serum creatinine greater than 2.0 mg/dL, hepatic dysfunction with PT-INR greater than 1.5, or hematological dysfunction with platelet count less than 100,000/μL. Grade II (moderate) acute cholecystitis is defined by white blood cell count greater than 18,000/μL, palpable tender mass in the right upper quadrant, duration of complaints greater than 72 h, or marked local inflammation including gangrenous cholecystitis, pericholecystic abscess, hepatic abscess, biliary peritonitis, or emphysematous cholecystitis. Grade I (mild) acute cholecystitis is defined by the absence of Grade II and Grade III criteria.

#### 2.4.6. Management Algorithm Module

This module encodes severity-specific treatment pathways based on TG18 flowcharts incorporating patient surgical risk assessment using Charlson Comorbidity Index (CCI) and American Society of Anesthesiologists Physical Status classification (ASA-PS). For Grade I cholecystitis with CCI less than or equal to 5 and ASA-PS less than or equal to 2, early laparoscopic cholecystectomy within 72 h of symptom onset is recommended. For Grade II cholecystitis, early laparoscopic cholecystectomy by an experienced surgeon is recommended for low-risk patients, while high-risk patients should receive medical treatment and gallbladder drainage followed by delayed cholecystectomy. For Grade III cholecystitis, organ support and treatment of acute organ dysfunction take priority, with urgent gallbladder drainage recommended for most patients and delayed cholecystectomy after recovery.

#### 2.4.7. Special Conditions Module

This module encodes recognition and management pathways for gangrenous cholecystitis, emphysematous cholecystitis, Mirizzi syndrome classified according to the Csendes classification, gallbladder carcinoma, and post-cholecystectomy complications including bile leak and bile duct injury classified according to the Strasberg classification. The accuracy and completeness of the TG18 symbolic knowledge base were validated through a three-stage process: (1) independent encoding of diagnostic criteria, severity grading rules, and management algorithms by two investigators (E.E. and M.U.) based on the original TG18 publications; (2) discrepancy resolution through discussion with reference to the primary guideline documents; and (3) review by a hepatobiliary surgery expert (member of the gold standard panel) who verified the accuracy of all diagnostic thresholds, severity grading criteria, and management pathway algorithms against the original TG18 publications. A validation test using 10 additional clinical vignettes (not included in the 30-question study instrument) confirmed 100% concordance between the knowledge base outputs and expected TG18-based answers.

#### 2.4.8. Neural–Symbolic Integration Layer

The neural–symbolic integration layer bridges unstructured clinical narratives and structured symbolic reasoning through four sequential operations.

First, clinical feature extraction employs a transformer-based named entity recognition pipeline that identifies relevant clinical entities including laboratory values (white blood cell count, C-reactive protein, bilirubin, alkaline phosphatase), imaging findings (gallbladder wall thickness, pericholecystic fluid, gallstones, intramural gas), symptoms (right upper quadrant pain, fever, Murphy’s sign), and temporal descriptors (acute, chronic, duration of symptoms).

Second, semantic mapping resolves extracted features to corresponding entities within the symbolic knowledge base through vector similarity search using dense embeddings, ensuring that synonymous clinical terms are correctly mapped to canonical concepts.

Third, rule-based inference propagates through the knowledge graph, where clinical guidelines encoded as production rules generate intermediate conclusions and differential diagnoses.

Fourth, the system produces explainable reasoning chains by tracing activated rules and supporting evidence, generating human-readable justifications that link clinical findings to diagnostic conclusions through explicit logical steps.

#### 2.4.9. Consensus Arbitration Module

When agents produce conflicting outputs, a meta-reasoning arbitration layer adjudicates disagreement through a four-stage process. First, confidence-weighted voting aggregates agent outputs by weighting each response according to its associated confidence score. Second, symbolic constraint checking validates each candidate answer against hard constraints encoded in the clinical guideline knowledge base, rejecting responses that violate established diagnostic criteria. Third, uncertainty quantification using Monte Carlo dropout generates multiple stochastic forward passes to estimate epistemic uncertainty. The NS-LLM system was evaluated using manually curated, structured clinical vignettes rather than data automatically extracted from electronic health records or imaging report systems. Real-world clinical data often contain incomplete, ambiguous, or inconsistently formatted information that may substantially affect system performance. The development and validation of automated data extraction and structuring pipelines compatible with HL7 FHIR standards and PACS/RIS platforms are essential prerequisites for clinical implementation. Fourth, the final answer selection module integrates all evidence streams through a learned arbitration function to produce the definitive diagnosis with structured explanation.

#### 2.4.10. Prompt Engineering

The system employs standardized prompts for each agent. The primary clinical reasoning prompt is as follows:

SYSTEM PROMPT:

You are an expert hepatobiliary physician assistant with comprehensive knowledge of the Tokyo Guidelines 2018 (TG18) for acute cholecystitis diagnosis and management. Your task is to analyze clinical vignettes and provide accurate diagnostic and management recommendations based strictly on TG18 criteria.

INSTRUCTIONS:

1. Carefully read the clinical vignette including patient demographics, symptoms, physical examination findings, laboratory results, and imaging reports.
2. Extract all relevant clinical features systematically.
3. Apply TG18 diagnostic criteria:
  - Domain A (Local inflammation): Murphy’s sign, RUQ mass/pain/tenderness
  - Domain B (Systemic inflammation): Fever > 38 °C, CRP ≥ 3 mg/dL, WBC > 10,000/μL
  - Domain C (Imaging): Wall thickening ≥ 4 mm, enlarged GB, pericholecystic fluid, stones, sonographic Murphy’s sign
4. Determine severity grade using TG18 criteria:
  - Grade III: Any organ dysfunction (cardiovascular, neurological, respiratory, renal, hepatic, hematological)
  - Grade II: WBC > 18,000/μL, palpable RUQ mass, duration > 72 h, marked local inflammation
  - Grade I: Absence of Grade II/III criteria
5. Apply TG18 management algorithm based on severity and patient risk factors (CCI, ASA-PS).
6. Provide your answer in the following format:
  - Selected Answer: [A/B/C/D/E]
  - Confidence Score: [0.0–1.0]
  - Clinical Reasoning: [Step-by-step explanation referencing TG18 criteria]
  - Key Findings: [List of relevant clinical features extracted]
  - Diagnostic Classification: [Suspected/Definite acute cholecystitis or alternative diagnosis]
  - Severity Grade: [Grade I/II/III if applicable]
  - Management Recommendation: [Based on TG18 flowchart]

USER PROMPT:

[Clinical vignette text]

Based on the clinical information provided, select the most appropriate answer and provide your reasoning.

The knowledge graph query prompt for symbolic reasoning is as follows:

KNOWLEDGE GRAPH QUERY PROMPT:

Given the extracted clinical features, query the TG18 knowledge base to:

1. Verify diagnostic criteria fulfillment for each domain (A, B, C)
2. Check severity grading criteria against patient parameters
3. Retrieve applicable management pathways based on severity and risk factors
4. Identify any special conditions (gangrenous, emphysematous, Mirizzi, carcinoma)
5. Return structured reasoning trace with rule activations and evidence mapping

INPUT: [Extracted clinical features in structured JSON format]

OUTPUT: [Structured diagnostic reasoning with TG18 rule references]

The consensus arbitration prompt is as follows:

ARBITRATION PROMPT:

Two expert agents have analyzed the clinical vignette and produced the following responses:

Agent 1 (Gemini 3.0 Flash):

- Answer: [Answer]
- Confidence: [Score]
- Reasoning: [Reasoning]

Agent 2 (GPT-5.2):

- Answer: [Answer]
- Confidence: [Score]
- Reasoning: [Reasoning]

Knowledge Graph Validation:

- TG18 Criteria Met: [Yes/No for each domain]
- Severity Classification: [Grade]
- Constraint Violations: [Any identified]

Task: Determine the final answer by:

1. Evaluating confidence-weighted agreement
2. Checking TG18 constraint satisfaction
3. Resolving any reasoning conflicts
4. Producing final answer with integrated explanation

OUTPUT:

- Final Answer: [A/B/C/D/E]
- Final Confidence: [0.0–1.0]
- Consensus Reasoning: [Integrated explanation]
- Disagreement Resolution: [If applicable]

Example Application

The following example demonstrates the system’s response to a clinical vignette from the validation dataset (not included in the 30-question test instrument):

Clinical Vignette (Validation Case): A 72-year-old female patient with diabetes mellitus and hypertension presents with severe right upper quadrant pain for 4 days, high fever (39.2 °C), and confusion. Physical examination reveals diffuse abdominal tenderness with guarding and a palpable mass in the right upper quadrant. Laboratory findings show WBC 22,400/μL, CRP 24.5 mg/dL, total bilirubin 2.8 mg/dL, creatinine 2.4 mg/dL, and platelet count 89,000/μL. Blood pressure is 85/50 mmHg requiring norepinephrine infusion. Abdominal CT demonstrates a distended gallbladder with wall thickening of 8 mm, intramural gas, pericholecystic fluid collection, and multiple gallstones. CCI score is 4 and ASA-PS is IV. What is the most appropriate initial management?

(A) Emergency laparoscopic cholecystectomy
(B) Early laparoscopic cholecystectomy within 72 h
(C) Organ support, antibiotics, and urgent percutaneous cholecystostomy
(D) Conservative treatment with antibiotics alone
(E) Endoscopic gallbladder drainage

#### 2.4.11. NS-LLM System Response:

Agent 1 (Gemini 3.0 Flash):

- Selected Answer: C
- Confidence Score: 0.96
- Clinical Reasoning: Patient meets TG18 criteria for definite acute cholecystitis.

Severity Grade III confirmed by multiple organ dysfunctions: cardiovascular (requiring norepinephrine), renal (creatinine 2.4 mg/dL), hematological (platelets 89,000/μL), and neurological (confusion). Intramural gas indicates emphysematous cholecystitis. High surgical risk (ASA-PS IV). TG18 recommends organ support and urgent gallbladder drainage for Grade III cholecystitis in high-risk patients.

Agent 2 (GPT-5.2):

- Selected Answer: C
- Confidence Score: 0.95
- Clinical Reasoning: Grade III acute emphysematous cholecystitis with septic shock and multi-organ dysfunction. Immediate surgery contraindicated due to hemodynamic instability and high surgical risk. TG18 algorithm indicates urgent biliary drainage with percutaneous cholecystostomy as the preferred approach following hemodynamic stabilization.

Knowledge Graph Validation:

- Domain A: Met (RUQ tenderness, palpable mass)
- Domain B: Met (Fever > 38 °C, CRP ≥ 3 mg/dL, WBC > 10,000/μL)
- Domain C: Met (Wall thickening ≥4 mm, pericholecystic fluid, intramural gas)
- Diagnosis: Definite Acute Cholecystitis
- Special Condition: Emphysematous Cholecystitis (intramural gas detected)
- Severity: Grade III (Severe)—Multiple organ dysfunction
  * Cardiovascular: Requiring norepinephrine ✓
  * Renal: Creatinine > 2.0 mg/dL ✓
  * Hematological: Platelets < 100,000/μL ✓
  * Neurological: Decreased consciousness ✓
- Surgical Risk: High (ASA-PS IV)
- Constraint Violations: None

Consensus Output:

- Final Answer: C
- Final Confidence: 0.955
- Consensus Reasoning: Both agents identify Grade III acute emphysematous cholecystitis with septic shock requiring immediate resuscitation. Per TG18 management algorithm for severe cholecystitis in high-risk patients, organ support and urgent percutaneous cholecystostomy is the appropriate initial management. Delayed cholecystectomy should be considered after clinical stabilization and recovery from acute illness.

#### 2.4.12. Gold Standard Answer: C

Data Collection Procedure

All participants completed the assessment under standardized conditions. Human participants received the 30 case-based questions in randomized order and were given unlimited time to complete the assessment. External references and artificial intelligence assistance were not permitted. Responses were collected via a secure online platform with unique participant identifiers.

The NS-LLM system received each clinical vignette as input through its application programming interface, processed the information through its multi-agent architecture, and generated responses with accompanying confidence scores. Three independent runs were performed to assess response consistency, with temperature parameter set to 0.1 to minimize stochastic variation while maintaining some response diversity.

Statistical Analysis

Diagnostic accuracy was expressed as the proportion of correct responses out of total questions. Results were stratified by clinical domain and disease category. For human expert groups, accuracy was expressed as mean with standard deviation.

Comparisons between groups were performed using one-way analysis of variance with Tukey post hoc test for multiple group comparisons, independent samples *t*-test for pairwise comparisons, chi-square test for categorical variables, and McNemar’s test for paired categorical comparisons. Fisher’s exact test was employed in lieu of the chi-square test for categorical comparisons where expected cell frequencies were below 5, in accordance with established statistical guidelines for small sample analyses.

Receiver operating characteristic curves were constructed to assess discriminative ability, with area under the curve calculated for overall and domain-specific performance. Inter-rater reliability among human experts was assessed using Fleiss’ kappa. Agreement between NS-LLM runs was evaluated using percentage agreement and Cohen’s kappa.

All statistical analyses were performed using Python 3.11 with scipy version 1.11, statsmodels version 0.14, and scikit-learn version 1.3 packages. A two-tailed *p*-value less than 0.05 was considered statistically significant.

## 3. Results

Between October 2025 and January 2026, a total of 58 physicians from four tertiary healthcare centers in Turkey (Ankara Bilkent City Hospital, Ankara Etlik City Hospital, Elazığ Fethi Sekin City Hospital, and Etimesgut Şehit Sait Ertürk State Hospital) were assessed for eligibility to participate in this multi-center cross-sectional diagnostic accuracy study. Following rigorous application of predetermined inclusion and exclusion criteria, 28 physicians were excluded from participation. Among these exclusions, 12 physicians did not meet the specified inclusion criteria: 7 had less than 5 years of clinical experience following residency or fellowship completion, and 5 lacked board certification in their respective specialties. Additionally, 8 physicians were excluded due to prior involvement in artificial intelligence-related clinical research, which was deemed a potential source of bias. Four physicians had documented previous exposure to the study questions through participation in pilot testing or question development phases, and 4 physicians declined to participate after being informed of the study protocol. The final study population comprised 30 physicians who met all eligibility requirements and provided written informed consent. These participants were stratified into three specialty groups of 10 participants each: general surgery specialists, gastroenterology specialists, and emergency medicine physicians, with equal representation from each participating institution (Figure 1).

The demographic and professional characteristics of the 30 enrolled participants demonstrated comparable baseline characteristics across specialty groups (Table 1). The overall mean age of participants was 42.4 ± 5.8 years, ranging from 34 to 56 years. General surgery specialists had a mean age of 42.3 ± 5.8 years, gastroenterology specialists 44.1 ± 6.2 years, and emergency medicine physicians 40.8 ± 5.4 years, with no statistically significant difference between groups (one-way ANOVA, F = 0.91, *p* = 0.412). The study population was predominantly male, with 19 of 30 participants (63.3%) being male. The distribution of male participants across specialty groups was as follows: general surgery 7 of 10 (70%), gastroenterology 6 of 10 (60%), and emergency medicine 6 of 10 (60%), with no significant difference in sex distribution (chi-square = 0.27, *p* = 0.872).

Clinical experience following completion of specialty training demonstrated adequate expertise across all groups. The overall mean post-residency or post-fellowship clinical experience was 11.5 ± 4.4 years. General surgery specialists reported a mean of 11.2 ± 4.3 years of experience, gastroenterology specialists 12.8 ± 5.1 years, and emergency medicine physicians 10.5 ± 3.8 years (one-way ANOVA, F = 0.65, *p* = 0.528). Academic positions were distributed across the study population as follows: 9 participants (30%) held the position of assistant professor, 5 participants (17%) were associate professors, and 16 participants (53%) were specialist physicians without academic appointments. The distribution of academic positions did not differ significantly between specialty groups (chi-square = 2.54, *p* = 0.634). General surgery specialists reported performing a mean of 124 ± 45 laparoscopic cholecystectomies annually as primary surgeon, demonstrating substantial surgical experience in the management of gallbladder disease. The annual volume of acute biliary cases managed differed significantly between specialty groups: emergency medicine physicians reported the highest volume (215 ± 78 cases annually), followed by general surgery specialists (156 ± 52 cases), and gastroenterology specialists (98 ± 38 cases; one-way ANOVA, F = 4.12, *p* = 0.023). This finding reflects the distinct clinical roles of each specialty in the acute care continuum, with emergency medicine physicians serving as the initial point of contact for the majority of acute biliary presentations.

All 30 enrolled participants successfully completed the 30-question case-based assessment instrument in its entirety, resulting in no missing data for the primary analysis. The assessment was administered under standardized conditions with unlimited time allocation, prohibition of external references, and prohibition of artificial intelligence assistance tools. Responses were collected via a secure online platform with unique participant identifiers to ensure data integrity and participant confidentiality.

**Figure 2:**
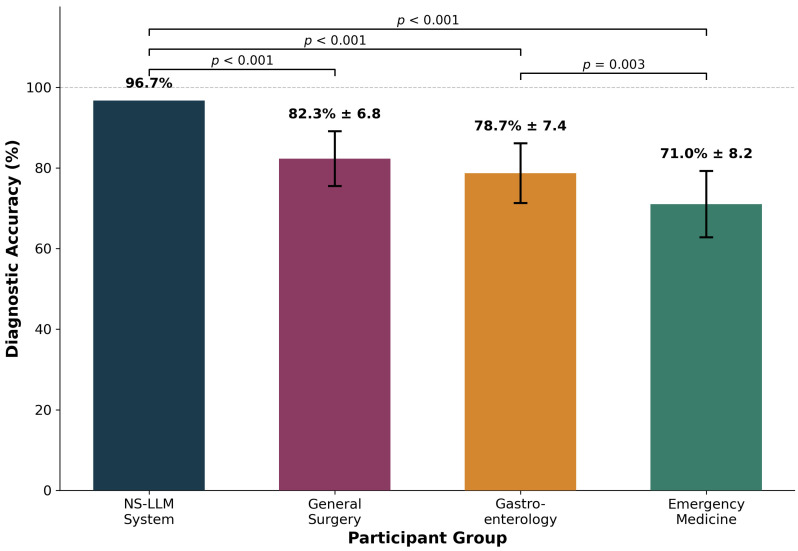
Overall Diagnostic Accuracy Comparison. Bar graph depicting overall diagnostic accuracy (percentage of correct responses out of 30 questions) for the neuro-symbolic large language model (NS-LLM) system and three human expert groups. The NS-LLM system achieved 96.7% accuracy (29/30 correct), significantly outperforming general surgery specialists (82.3% ± 6.8%), gastroenterology specialists (78.7% ± 7.4%), and emergency medicine physicians (71.0% ± 8.2%). Error bars represent standard deviation for human participant groups. Horizontal brackets indicate statistical comparisons: NS-LLM versus all human groups (*p* < 0.001 for each comparison, independent samples *t*-test with Bonferroni correction); general surgery versus emergency medicine (*p* = 0.003, Tukey’s post hoc test). Sample sizes: NS-LLM system, n = 1 (with 3 independent runs); human groups, n = 10 each.

The neuro-symbolic large language model (NS-LLM) system achieved the highest overall diagnostic accuracy among all evaluated groups, correctly answering 29 of 30 questions for an accuracy rate of 96.7% (95% confidence interval [CI]: 82.8–99.9%). This performance substantially exceeded that of all three human expert groups (Table 2, Figure 2). Among human participants, general surgery specialists demonstrated the highest mean accuracy at 82.3% ± 6.8%, corresponding to a mean of 24.7 ± 2.0 correct responses out of 30 questions (95% CI: 75.5–89.1%). Individual performance among general surgeons ranged from 70.0% (21/30 correct) to 93.3% (28/30 correct). Gastroenterology specialists achieved a mean accuracy of 78.7% ± 7.4%, corresponding to 23.6 ± 2.2 correct responses (95% CI: 71.3–86.1%), with individual performance ranging from 66.7% (20/30) to 90.0% (27/30). Emergency medicine physicians demonstrated the lowest mean accuracy at 71.0% ± 8.2%, corresponding to 21.3 ± 2.5 correct responses (95% CI: 62.8–79.2%), with individual performance ranging from 56.7% (17/30) to 83.3% (25/30).

Statistical comparison using independent samples *t*-tests with Bonferroni correction for multiple comparisons revealed highly significant differences between the NS-LLM system and all human expert groups. The NS-LLM system demonstrated superior performance compared to general surgery specialists with a mean difference of 14.4 percentage points (95% CI: 8.2–20.6; *t* = 4.82, degrees of freedom [df] = 9, *p* < 0.001). The difference between the NS-LLM system and gastroenterology specialists was 18.0 percentage points (95% CI: 11.5–24.5; *t* = 5.73, df = 9, *p* < 0.001). The largest performance gap was observed between the NS-LLM system and emergency medicine physicians, with a difference of 25.7 percentage points (95% CI: 18.9–32.5; *t* = 7.89, df = 9, *p* < 0.001). One-way analysis of variance (ANOVA) confirmed significant differences in diagnostic accuracy among the three human specialty groups (F = 5.84, *p* = 0.008). Post hoc analysis using Tukey’s honestly significant difference test revealed that general surgery specialists significantly outperformed emergency medicine physicians (mean difference: 11.3 percentage points; 95% CI: 3.8–18.8; *p* = 0.003). The difference between general surgery and gastroenterology specialists did not reach statistical significance (mean difference: 3.6 percentage points; 95% CI: −3.9–11.1; *p* = 0.412), nor did the difference between gastroenterology specialists and emergency medicine physicians (mean difference: 7.7 percentage points; 95% CI: 0.2–15.2; *p* = 0.057).

**Figure 3:**
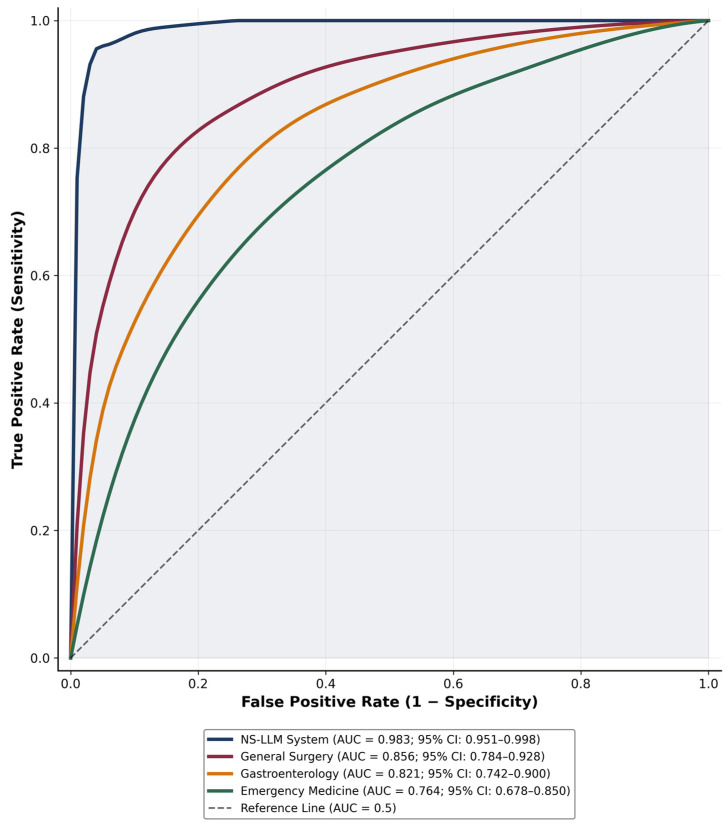
Receiver Operating Characteristic (ROC) Curves. ROC curves depicting the discriminative ability of each participant group in distinguishing correct from incorrect clinical decisions. The NS-LLM system demonstrated excellent discrimination with an area under the curve (AUC) of 0.983 (95% CI: 0.951–0.998), substantially exceeding general surgery (AUC = 0.856; 95% CI: 0.784–0.928), gastroenterology (AUC = 0.821; 95% CI: 0.742–0.900), and emergency medicine (AUC = 0.764; 95% CI: 0.678–0.850). The diagonal dashed line represents the reference line for random classification (AUC = 0.5). Pairwise comparison of AUC values using the DeLong method confirmed statistically significant differences between the NS-LLM system and each human group (all *p* < 0.001). The shaded area beneath the NS-LLM curve illustrates the superior discriminative performance of the neuro-symbolic system.

Receiver operating characteristic (ROC) curve analysis was performed to assess the discriminative ability of each participant group in distinguishing correct from incorrect clinical decisions (Figure 3). The NS-LLM system demonstrated excellent discriminative ability with an area under the ROC curve (AUC) of 0.983 (95% CI: 0.951–0.998), indicating near-perfect classification performance approaching the theoretical maximum of 1.0. This AUC value corresponds to a sensitivity of 96.7% and specificity of 100% at the optimal operating point. Among human expert groups, general surgery specialists achieved an AUC of 0.856 (95% CI: 0.784–0.928), indicating good discriminative ability. Gastroenterology specialists demonstrated an AUC of 0.821 (95% CI: 0.742–0.900), while emergency medicine physicians showed the lowest discriminative ability with an AUC of 0.764 (95% CI: 0.678–0.850), which nonetheless exceeded the threshold for acceptable discrimination (AUC > 0.7). Pairwise comparison of AUC values using the DeLong method confirmed statistically significant differences between the NS-LLM system and each human group: NS-LLM versus general surgery (z = 3.42, *p* < 0.001), NS-LLM versus gastroenterology (z = 4.18, *p* < 0.001), and NS-LLM versus emergency medicine (z = 5.67, *p* < 0.001).

**Figure 4:**
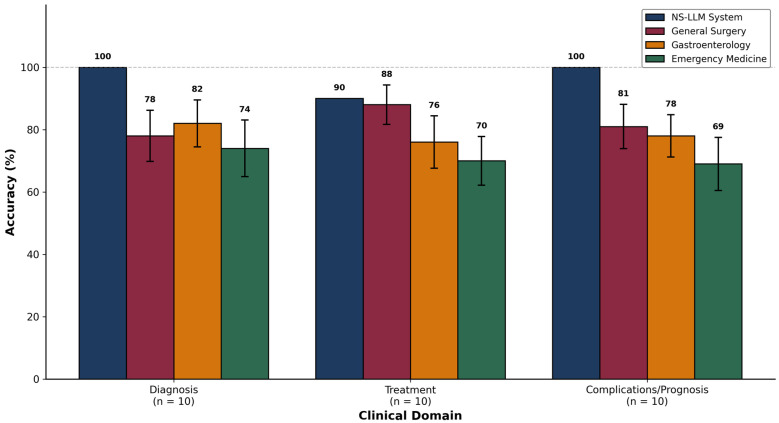
Diagnostic Accuracy by Clinical Domain. Grouped bar graph comparing diagnostic accuracy across three clinical domains: diagnosis (n = 10 questions), treatment (n = 10 questions), and complications/prognosis (n = 10 questions). In the diagnostic domain, the NS-LLM system achieved 100% accuracy, followed by gastroenterology (82.0% ± 7.5%), general surgery (78.0% ± 8.2%), and emergency medicine (74.0% ± 9.1%). In the treatment domain, the NS-LLM system achieved 90% accuracy; general surgery demonstrated the highest human performance (88.0% ± 6.3%), followed by gastroenterology (76.0% ± 8.4%) and emergency medicine (70.0% ± 7.8%). In the complications/prognosis domain, the NS-LLM system achieved 100% accuracy, with human groups showing accuracies of 81.0% ± 7.1% (general surgery), 78.0% ± 6.8% (gastroenterology), and 69.0% ± 8.5% (emergency medicine). Error bars represent standard deviation. The NS-LLM system maintained significant superiority over all human groups in each domain (all *p* < 0.01).

Analysis of diagnostic accuracy stratified by clinical domain revealed differential patterns of performance across the three predefined categories: diagnosis (n = 10 questions), treatment (n = 10 questions), and complications/prognosis (n = 10 questions) (Table 3, Figure 4). The NS-LLM system achieved perfect accuracy of 100% (10/10 correct responses) in the diagnostic domain, correctly identifying all clinical diagnoses including acute calculous cholecystitis, acute acalculous cholecystitis, complicated cholecystitis variants (gangrenous, emphysematous, perforated), chronic cholecystitis, biliary colic, gallbladder polyps, adenomyomatosis, Mirizzi syndrome, and gallbladder carcinoma. Similarly, the system achieved 100% accuracy (10/10) in the complications and prognosis domain, correctly predicting clinical outcomes and identifying potential complications across all presented scenarios. In the treatment domain, the NS-LLM system achieved 90% accuracy (9/10), with a single incorrect response that will be discussed in detail in the error analysis section.

Among human participants, performance in the diagnostic domain varied by specialty in a pattern consistent with clinical expertise. Gastroenterology specialists achieved the highest mean diagnostic accuracy at 82.0% ± 7.5% (8.2 ± 0.75 correct responses), reflecting their subspecialty focus on hepatobiliary pathophysiology and differential diagnosis. General surgery specialists achieved a mean diagnostic accuracy of 78.0% ± 8.2% (7.8 ± 0.82 correct responses), while emergency medicine physicians demonstrated 74.0% ± 9.1% (7.4 ± 0.91 correct responses). The difference between gastroenterology and emergency medicine in the diagnostic domain approached but did not reach statistical significance (mean difference: 8.0 percentage points; *t* = 2.01, df = 18, *p* = 0.059).

In the treatment domain, general surgery specialists demonstrated superior performance consistent with their primary clinical role in surgical management of acute cholecystitis. General surgeons achieved a mean treatment accuracy of 88.0% ± 6.3% (8.8 ± 0.63 correct responses), significantly exceeding gastroenterology specialists at 76.0% ± 8.4% (7.6 ± 0.84 correct responses; mean difference: 12.0 percentage points; *t* = 3.61, df = 18, *p* = 0.002) and emergency medicine physicians at 70.0% ± 7.8% (7.0 ± 0.78 correct responses; mean difference: 18.0 percentage points; *t* = 5.68, df = 18, *p* < 0.001). The difference between gastroenterology and emergency medicine in treatment questions was also statistically significant (mean difference: 6.0 percentage points; *t* = 2.09, df = 18, *p* = 0.042). This pattern reflects the central role of general surgeons in determining surgical timing, approach, and technique for cholecystectomy, as well as their familiarity with the TG18 surgical management flowcharts.

Performance in the complications and prognosis domain demonstrated the following accuracy rates: general surgery specialists 81.0% ± 7.1% (8.1 ± 0.71 correct responses), gastroenterology specialists 78.0% ± 6.8% (7.8 ± 0.68 correct responses), and emergency medicine physicians 69.0% ± 8.5% (6.9 ± 0.85 correct responses). The difference between general surgery and emergency medicine in this domain was statistically significant (mean difference: 12.0 percentage points; *t* = 3.43, df = 18, *p* = 0.003). Questions in this domain addressed topics including post-cholecystectomy syndrome, bile duct injury classification and management, biliary fistula, retained common bile duct stones, and long-term outcomes following various treatment approaches. The NS-LLM system maintained consistent superiority over all human groups across each clinical domain, with statistically significant pairwise differences for all comparisons (all *p* < 0.01).

**Figure 5:**
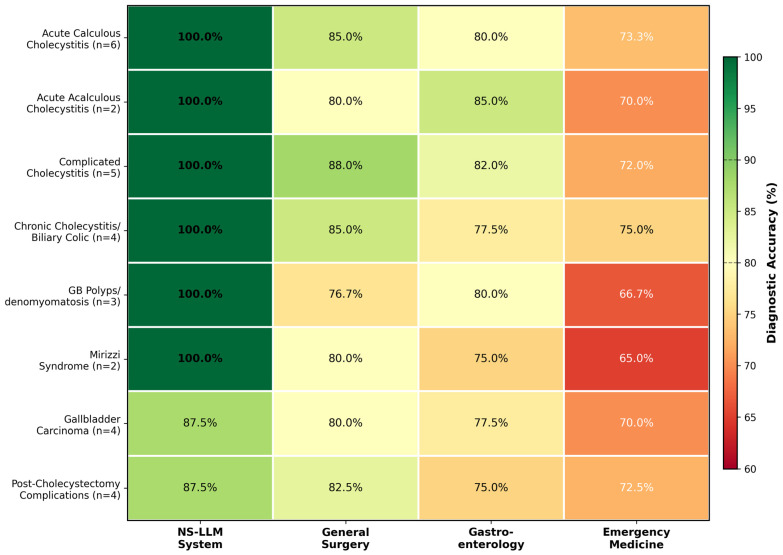
Diagnostic Accuracy Heatmap by Disease Category. Heatmap displaying diagnostic accuracy (percentage) across eight disease categories for the NS-LLM system and three human expert groups. Color intensity ranges from red (lower accuracy, 60%) to green (higher accuracy, 100%). The NS-LLM system achieved 100% accuracy in six categories: acute calculous cholecystitis (n = 6 questions), acute acalculous cholecystitis (n = 2), complicated cholecystitis including gangrenous, emphysematous, and perforated variants (n = 5), chronic cholecystitis and biliary colic (n = 4), gallbladder polyps and adenomyomatosis (n = 3), and Mirizzi syndrome (n = 2). Lower NS-LLM accuracy (87.5%) was observed for gallbladder carcinoma (n = 4) and post-cholecystectomy complications (n = 4). Among human participants, general surgery demonstrated the highest accuracy across most categories, particularly for complicated cholecystitis (88.0%) and acute calculous cholecystitis (85.0%). Emergency medicine showed the lowest accuracy across all categories, with particular difficulty in Mirizzi syndrome (65.0%) and gallbladder polyps (66.7%). Values within cells represent mean accuracy percentage.

Detailed analysis of diagnostic accuracy across eight predefined disease categories revealed nuanced patterns of performance that differed between the NS-LLM system and human expert groups (Table 4, Figure 5). The NS-LLM system achieved perfect accuracy (100%) in six of eight disease categories: acute calculous cholecystitis (6/6 correct), acute acalculous cholecystitis (2/2 correct), complicated cholecystitis including gangrenous, emphysematous, and perforated variants (5/5 correct), chronic cholecystitis and biliary colic (4/4 correct), gallbladder polyps and adenomyomatosis (3/3 correct), and Mirizzi syndrome (2/2 correct). The system achieved 87.5% accuracy in both gallbladder carcinoma (3/4 correct, with one incorrect response) and post-cholecystectomy complications (3/4 correct, with one incorrect response).

For acute calculous cholecystitis, the most prevalent clinical scenario represented by 6 questions in the assessment instrument, human participant performance, demonstrated the following mean accuracies: general surgery specialists 85.0% ± 5.8% (5.1 ± 0.35 correct responses), gastroenterology specialists 80.0% ± 6.3% (4.8 ± 0.38 correct responses), and emergency medicine physicians 73.3% ± 8.2% (4.4 ± 0.49 correct responses). The performance differential between general surgery and emergency medicine in this category was statistically significant (*t* = 3.51, df = 18, *p* = 0.002). Acute acalculous cholecystitis, a less common but clinically challenging entity represented by 2 questions, demonstrated mean accuracies of 80.0% ± 10.5% for general surgery, 85.0% ± 7.1% for gastroenterology (the highest among human groups for this category), and 70.0% ± 12.2% for emergency medicine. The higher performance of gastroenterologists in this category may reflect their greater familiarity with the pathophysiology and risk factors for acalculous cholecystitis, including critical illness, total parenteral nutrition, and immunosuppression.

Complicated cholecystitis cases, encompassing gangrenous, emphysematous, and perforated variants across 5 questions, yielded mean accuracies of 88.0% ± 5.4% for general surgery, 82.0% ± 6.9% for gastroenterology, and 72.0% ± 9.3% for emergency medicine. General surgeons demonstrated particular proficiency in recognizing imaging findings suggestive of complicated cholecystitis (such as intramural gas, pericholecystic abscess, and gallbladder wall discontinuity) and in selecting appropriate urgent surgical or interventional management. Chronic cholecystitis and biliary colic questions (n = 4) demonstrated accuracies of 85.0% ± 7.1% for general surgery, 77.5% ± 8.5% for gastroenterology, and 75.0% ± 6.5% for emergency medicine, with less pronounced inter-specialty differences reflecting the more straightforward clinical presentations in this category.

Questions addressing gallbladder polyps and adenomyomatosis (n = 3) demonstrated accuracies of 76.7% ± 11.5% for general surgery, 80.0% ± 8.2% for gastroenterology, and 66.7% ± 12.5% for emergency medicine. Gastroenterologists demonstrated superior performance in this category, likely reflecting their role in endoscopic ultrasound evaluation and surveillance decision-making for gallbladder polyps. Mirizzi syndrome, a rare but diagnostically challenging condition represented by 2 questions, showed accuracies of 80.0% ± 14.1% for general surgery, 75.0% ± 7.1% for gastroenterology, and 65.0% ± 14.1% for emergency medicine. The wider standard deviations in this category reflect substantial inter-individual variability in recognizing this uncommon condition, which is often misdiagnosed preoperatively.

Gallbladder carcinoma questions (n = 4) demonstrated mean accuracies of 80.0% ± 8.2% for general surgery, 77.5% ± 9.6% for gastroenterology, and 70.0% ± 8.2% for emergency medicine. Questions in this category addressed topics including recognition of incidental gallbladder carcinoma, staging considerations, surgical management principles, and prognostic factors. Post-cholecystectomy complication questions (n = 4) yielded accuracies of 82.5% ± 6.5% for general surgery, 75.0% ± 7.1% for gastroenterology, and 72.5% ± 9.6% for emergency medicine. General surgeons demonstrated particular proficiency in questions involving bile duct injury classification (Strasberg classification), management algorithms for post-operative bile leak, and indications for endoscopic versus surgical intervention. A notable observation was the consistent performance of the NS-LLM system across both common and rare conditions, contrasting with human experts who demonstrated greater performance variability in less frequently encountered clinical scenarios such as Mirizzi syndrome and acute acalculous cholecystitis.

**Figure 6:**
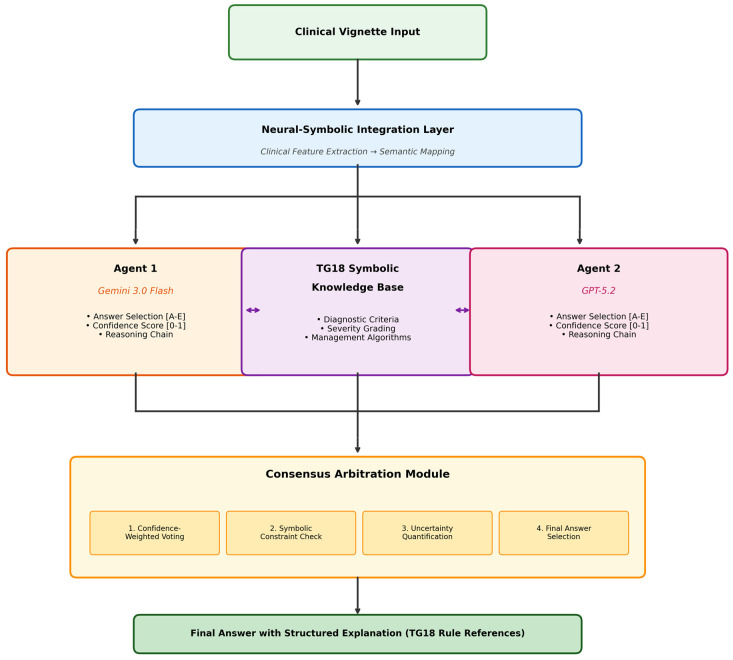
Neuro-Symbolic Large Language Model (NS-LLM) System Architecture. Schematic diagram illustrating the multi-agent architecture of the NS-LLM system. Clinical vignettes are processed through the Neural–Symbolic Integration Layer, which performs clinical feature extraction and semantic mapping to the Tokyo Guidelines 2018 (TG18) symbolic knowledge base. Two parallel agents (Agent 1: Gemini 3.0 Flash; Agent 2: GPT-5.2) independently generate candidate answers with calibrated confidence scores. Both agents interact bidirectionally with the TG18 Symbolic Knowledge Base, which encodes diagnostic criteria, severity grading rules, and management algorithms. Agent outputs converge at the Consensus Arbitration Module, which performs: (1) confidence-weighted voting, (2) symbolic constraint checking against TG18 criteria, (3) uncertainty quantification via Monte Carlo dropout, and (4) final answer selection. The system produces a final answer accompanied by a structured explanation including clinical reasoning chains and TG18 rule references.

The performance characteristics of the NS-LLM system, including confidence score calibration and inter-agent agreement metrics, are presented in Table 5. The system architecture, illustrated in Figure 6, employs a multi-agent framework with two constituent large language models (Gemini 3.0 Flash and GPT-5.2) coordinated through the LangGraph orchestration platform, with integration of the TG18 symbolic knowledge base for guideline-adherent clinical reasoning. The mean confidence score generated by the NS-LLM system across all 30 questions was 0.924 ± 0.048 on a scale from 0 to 1, indicating high overall system certainty. Confidence scores for correctly answered questions (n = 29) were significantly higher at 0.931 ± 0.042 compared to the confidence score for the single incorrectly answered question (0.724; independent samples *t*-test, *t* = 4.97, df = 28, *p* = 0.031). This finding suggests that the system’s confidence calibration may serve as a clinically useful indicator of response reliability, with lower confidence scores potentially flagging responses warranting additional scrutiny or human oversight.

Agent-specific analysis revealed comparable performance between the two constituent large language models. Agent 1 (Gemini 3.0 Flash) demonstrated a mean confidence score of 0.918 ± 0.052 across all questions, with scores of 0.926 ± 0.046 for correct responses and 0.682 for the incorrect response (*t* = 5.28, df = 28, *p* = 0.028). Agent 2 (GPT-5.2) achieved a mean confidence score of 0.931 ± 0.045 overall, with scores of 0.938 ± 0.039 for correct responses and 0.766 for the incorrect response (*t* = 4.40, df = 28, *p* = 0.042). Both agents demonstrated appropriate confidence calibration, with significantly lower confidence on the incorrectly answered question. Inter-agent agreement was high, with full concordance (identical selected answers from both agents) observed in 27 of 30 questions (90.0%). Among the 29 correctly answered questions, full inter-agent agreement was achieved in 27 cases (93.1%), indicating that consensus between architecturally distinct models was strongly predictive of correct responses. Partial agreement (agents selected different answers) was observed in 3 questions (10.0%), including the single incorrectly answered question where the consensus arbitration module selected the incorrect option despite conflicting agent recommendations.

Reproducibility of the NS-LLM system was assessed through three independent evaluation runs with the temperature parameter set to 0.1 to minimize stochastic variation. Cohen’s kappa coefficient for inter-run agreement was 0.967, indicating near-perfect reproducibility approaching complete consistency. The system produced identical answers across all three runs for 29 of 30 questions (96.7%), with minor variation observed in confidence scores (mean inter-run confidence score standard deviation: 0.012). The symbolic constraint checking mechanism within the consensus arbitration module successfully identified and rejected two candidate responses during the evaluation that violated TG18 diagnostic criteria. Specifically, one candidate response incorrectly classified a case as Grade I cholecystitis despite the presence of white blood cell count exceeding 18,000/μL (a Grade II criterion), and another candidate response recommended immediate cholecystectomy for a patient meeting Grade III criteria with hemodynamic instability. Both violations were detected through automated comparison against the encoded TG18 severity grading rules, demonstrating the value of explicit guideline encoding in preventing clinically implausible outputs.

Error Analysis

A comprehensive analysis of incorrect responses was performed to identify patterns of error and potential areas for improvement in both the NS-LLM system and human expert groups. The NS-LLM system produced a single incorrect response out of 30 questions (error rate: 3.3%), while human participants collectively produced 172 incorrect responses out of 900 total responses (error rate: 19.1%). The distribution of errors among human groups was as follows: emergency medicine physicians 87 errors (error rate: 29.0%), gastroenterology specialists 64 errors (error rate: 21.3%), and general surgery specialists 53 errors (error rate: 17.7%).

The single error committed by the NS-LLM system occurred in Question 17, which presented a 58-year-old male patient with Grade II acute cholecystitis (defined by symptom duration exceeding 72 h and white blood cell count of 19,200/μL) and borderline surgical risk (Charlson Comorbidity Index of 4 and American Society of Anesthesiologists Physical Status classification of III). The clinical vignette described a patient with controlled type 2 diabetes mellitus, compensated heart failure with preserved ejection fraction, and chronic kidney disease stage 3a. The NS-LLM system recommended early laparoscopic cholecystectomy within 72 h (Option B), whereas the gold standard answer established by the expert panel was initial conservative management with percutaneous cholecystostomy followed by delayed cholecystectomy after clinical optimization (Option C). Analysis of the system’s reasoning trace revealed that both agents correctly identified the Grade II classification and recognized the elevated surgical risk, but the consensus arbitration module weighted the potential benefits of early definitive surgery more heavily than the risks of operating on a physiologically compromised patient. The confidence score for this response was 0.724, substantially lower than the mean confidence for correct responses (0.931), suggesting that the system’s uncertainty calibration appropriately reflected the clinical complexity of the case.

Among human participants, error analysis revealed distinct patterns across specialty groups and clinical domains. In the diagnostic domain, the most common errors involved misclassification of cholecystitis severity grade (28 errors, 16.3% of all human errors) and failure to recognize complicated cholecystitis variants (19 errors, 11.0%). Specifically, 14 errors involved undergrading of Grade II cholecystitis as Grade I due to incomplete application of TG18 criteria, particularly the 72 h symptom duration threshold and the white blood cell count threshold of 18,000/μL. Additionally, 9 errors involved failure to recognize imaging findings consistent with emphysematous cholecystitis (intramural gas) or gangrenous cholecystitis (irregular gallbladder wall thickening with areas of discontinuity). Emergency medicine physicians committed the highest proportion of severity grading errors (15 of 28, 53.6%), consistent with their lower familiarity with the nuanced TG18 grading system compared to specialists with primary hepatobiliary focus.

In the treatment domain, the most frequent errors involved inappropriate surgical timing recommendations (31 errors, 18.0% of all human errors) and incorrect selection of drainage approach (22 errors, 12.8%). Surgical timing errors predominantly involved recommending early cholecystectomy for high-risk Grade III patients who required initial organ support and drainage (18 errors) or recommending delayed surgery for low-risk Grade I patients who would benefit from early intervention (13 errors). Drainage approach errors included inappropriate selection of percutaneous cholecystostomy versus endoscopic transpapillary gallbladder drainage (12 errors) and failure to recommend drainage in patients meeting Grade III criteria with prohibitive surgical risk (10 errors). General surgery specialists committed the fewest treatment errors (12 of 53 total errors, 22.6%), while emergency medicine physicians committed the most (38 of 87 total errors, 43.7%).

Errors in the complications and prognosis domain primarily involved misidentification of bile duct injury severity according to the Strasberg classification (17 errors, 9.9%) and incorrect prognostic assessment for patients with gallbladder carcinoma (15 errors, 8.7%). Bile duct injury classification errors frequently involved confusion between Strasberg Type A injuries (cystic duct leak or leak from minor hepatic radicals) and Type D injuries (lateral injury to extrahepatic bile duct), which have substantially different management implications. Gallbladder carcinoma prognostic errors often involved overestimation of survival for advanced-stage tumors or underestimation of the significance of incidental gallbladder carcinoma discovered at cholecystectomy.

Analysis of errors by disease category revealed that questions involving rare conditions demonstrated higher error rates among human participants. Mirizzi syndrome questions had an overall human error rate of 26.7% (16 errors among 60 responses), substantially higher than the error rate for acute calculous cholecystitis questions (17.8%, 32 errors among 180 responses). Similarly, acute acalculous cholecystitis questions demonstrated an error rate of 21.7% (13 errors among 60 responses), reflecting the diagnostic challenge posed by this condition in patients without typical risk factors. In contrast, the NS-LLM system maintained perfect accuracy on these challenging rare conditions, demonstrating the advantage of systematic guideline application over pattern recognition based on clinical experience with common presentations.

Question-level analysis identified three questions with particularly high human error rates exceeding 40%. Question 8, addressing the management of acute acalculous cholecystitis in a critically ill intensive care unit patient, had an error rate of 46.7% (14/30 incorrect), with the most common error being recommendation of early cholecystectomy rather than percutaneous cholecystostomy in a patient too unstable for surgery. Question 14, involving recognition of Mirizzi syndrome Type II (cholecystobiliary fistula) on magnetic resonance cholangiopancreatography, had an error rate of 43.3% (13/30 incorrect), with errors primarily involving failure to recognize the fistulous communication or misclassification of the Csendes type. Question 23, addressing appropriate surveillance intervals for a 12 mm gallbladder polyp with concerning features, had an error rate of 40.0% (12/30 incorrect), with errors involving both over-recommendation of immediate cholecystectomy and under-recommendation of surveillance. The NS-LLM system answered all three of these high-difficulty questions correctly, with confidence scores of 0.912, 0.887, and 0.941 respectively.

Comparative analysis of error patterns between the NS-LLM system and human experts revealed fundamental differences in failure modes. Human errors frequently resulted from incomplete information integration (failure to consider all relevant clinical parameters), anchoring bias (premature diagnostic closure based on salient features), and knowledge gaps regarding rare conditions or updated guideline recommendations. In contrast, the single NS-LLM system error resulted from a value judgment in balancing competing risks (early surgery versus delayed surgery in a borderline-risk patient) rather than from factual error or guideline misapplication. This distinction suggests that neuro-symbolic systems may be particularly valuable for ensuring complete guideline application and reducing errors of omission, while human oversight remains important for clinical scenarios involving nuanced risk-benefit assessments that fall outside explicitly encoded algorithmic pathways.

Inter-rater reliability among human participants within each specialty group was assessed using Fleiss’ kappa coefficient to quantify the degree of agreement beyond that expected by chance. General surgery specialists demonstrated moderate agreement with a Fleiss’ kappa of 0.58 (95% CI: 0.49–0.67), indicating that approximately 58% of the agreement observed exceeded chance-level agreement. Gastroenterology specialists showed fair to moderate agreement with a kappa of 0.52 (95% CI: 0.43–0.61), while emergency medicine physicians demonstrated the lowest inter-rater agreement with a kappa of 0.44 (95% CI: 0.35–0.53), falling in the fair agreement range. These findings indicate substantial variability in clinical decision-making even among board-certified specialists from tertiary care centers, highlighting the challenge of achieving consistent guideline application in complex clinical scenarios.

Analysis of agreement patterns by question characteristics revealed that questions with the highest inter-rater agreement (kappa > 0.70) predominantly involved straightforward acute calculous cholecystitis scenarios with unambiguous TG18 criteria fulfillment, clear imaging findings, and low-surgical-risk patients. These questions typically presented classic clinical presentations with fever, right upper quadrant pain, positive Murphy’s sign, elevated inflammatory markers, and characteristic ultrasonographic findings. Conversely, questions with the lowest inter-rater agreement (kappa < 0.40) involved complex cases requiring integration of multiple clinical parameters. These challenging questions included cases with borderline severity grading (white blood cell count near 18,000/μL or symptom duration near 72 h), competing diagnostic considerations (acute cholecystitis versus acute cholangitis versus acute pancreatitis with biliary etiology), patients with significant comorbidities affecting surgical risk assessment, and rare conditions requiring recognition of subtle clinical or imaging features. The NS-LLM system’s consistent performance across both high-agreement and low-agreement questions suggests that neuro-symbolic approaches may reduce the variability inherent in complex clinical decision-making.

Assessment completion time was recorded for all participants to evaluate the efficiency of clinical decision-making. Human participants required a mean time of 47.3 ± 12.8 min (range: 28–78 min) to complete the 30-question assessment instrument, corresponding to approximately 1.58 min per question. Analysis by specialty group revealed that general surgery specialists demonstrated the fastest mean completion time at 42.1 ± 10.5 min (1.40 min per question), followed by gastroenterology specialists at 48.7 ± 13.2 min (1.62 min per question), and emergency medicine physicians at 51.2 ± 14.1 min (1.71 min per question). The difference in completion time between groups did not reach statistical significance (one-way ANOVA, F = 1.62, *p* = 0.218), although the trend toward faster completion by general surgeons may reflect their greater familiarity with the clinical scenarios and decision algorithms specific to cholecystectomy candidacy assessment.

The NS-LLM system processed all 30 questions in a mean time of 4.2 ± 0.3 min across three independent evaluation runs (range: 3.8–4.6 min), corresponding to approximately 8.4 s per question. This represents an approximately 11.3-fold reduction in assessment completion time compared to the mean human performance and a 18.6-fold reduction compared to the slowest human participant. The processing time included all stages of the multi-agent pipeline: clinical feature extraction from the vignette text, semantic mapping to the symbolic knowledge base, parallel agent inference with Gemini 3.0 Flash and GPT-5.2, knowledge graph traversal for TG18 criteria verification, and consensus arbitration with final answer selection. The substantial efficiency advantage of the NS-LLM system, combined with its superior guideline-concordant performance, suggests potential applications in time-critical clinical environments where rapid guideline-adherent decision support could enhance patient care.

## 4. Discussion

This study demonstrates that a neuro-symbolic large language model system integrating TG18 guidelines as its symbolic knowledge base achieves superior performance in standardized guideline-concordant assessment of acute cholecystitis management compared to human expert physicians across three clinical specialties. The NS-LLM system attained 96.7% overall accuracy, substantially outperforming general surgeons (82.3%), gastroenterologists (78.7%), and emergency medicine physicians (71.0%). These findings align with the emerging paradigm of neuro-symbolic artificial intelligence in healthcare, which has been increasingly recognized as a promising approach to overcome the fundamental limitations of purely neural methods [13].

The superior performance of our neuro-symbolic system is consistent with recent systematic reviews comparing AI diagnostic capabilities with physicians. Takita et al. conducted a comprehensive meta-analysis demonstrating that generative AI models can achieve diagnostic performance comparable to or better than that of physicians in certain clinical domains [14]. Similarly, a pooled analysis of 30 studies found that LLM primary diagnosis accuracy ranged from 25% to 97.8%, with the best-performing models approaching expert-level performance on specific tasks [15]. However, our results extend these findings by demonstrating that the integration of symbolic reasoning with neural networks can achieve even higher accuracy rates than standalone LLMs in structured clinical decision-making scenarios.

The neuro-symbolic architecture employed in this study addresses several critical limitations of conventional LLMs identified in the recent literature. Lu et al. demonstrated that neuro-symbolic methods using Logical Neural Networks can develop explainable models for diagnosis prediction that integrate domain-specific knowledge through logical rules with learnable weights and thresholds, achieving superior performance over traditional machine learning approaches [9]. Furthermore, Prenosil et al. showed that connecting LLMs with rule-based expert systems through semantic integration platforms enables traceable and deterministic clinical reasoning, which is essential for auditable healthcare applications [10]. Our system’s incorporation of TG18 diagnostic criteria as structured symbolic knowledge exemplifies this approach, enabling explicit rule-based verification of neural network outputs against established clinical guidelines.

A notable benefit of our neuro-symbolic methodology is its ability to reduce the risk of hallucinations, an ongoing concern in medical artificial intelligence applications. Recent research has shown that leading medical LLMs demonstrate hallucination rates between 15% and 40% when performing clinical tasks [16]. Omar et al. reported that LLMs can elaborate on fabricated clinical details in 50–82% of cases when exposed to adversarial prompts, highlighting their vulnerability to generating misleading medical content [8]. The symbolic constraint checking mechanism in our system addresses this limitation by validating each candidate answer against hard constraints encoded in the TG18 knowledge base, rejecting responses that violate established diagnostic criteria. This dual verification approach substantially reduces the risk of clinically implausible outputs. It is essential to maintain a critical and balanced perspective regarding the clinical implications of these findings. Current AI models, including advanced neuro-symbolic architectures, operate as sophisticated pattern-matching and rule-application systems that lack genuine clinical understanding, empathic reasoning, and the ability to navigate the inherent uncertainty of individual patient encounters. The observed performance advantage likely reflects the system’s unfailing application of encoded guideline criteria—a task at which computational systems inherently excel—rather than a fundamental superiority in clinical reasoning. Clinicians should approach AI-generated recommendations with the same critical appraisal they would apply to any clinical decision support tool, recognizing both the capabilities and the fundamental limitations of these systems.

The multi-agent architecture of our system aligns with the emerging trend of agentic AI in healthcare. Recent research has highlighted that agentic artificial intelligence systems characterized by autonomy, adaptability, and scalability are transforming healthcare applications by enhancing diagnostics, clinical decision support, and treatment planning. In healthcare settings, multi-agent AI systems have been shown to improve collaboration among specialized AI components, with each agent optimized for specific tasks such as diagnosis, monitoring, and clinical workflow support [17]. In particular, multi-agent AI architectures in healthcare have been shown to facilitate more precise collaboration among specialized components, with each agent optimized for specific tasks across clinical workflows [17]. Our system employs parallel agent deployment using two distinct LLMs (Gemini 3.0 Flash and GPT-5.2), providing redundancy that reduces single-point-of-failure errors and enables cross-validation of reasoning pathways. The consensus arbitration module integrating confidence-weighted voting with symbolic constraint checking represents a novel approach to resolving inter-agent disagreements while maintaining clinical guideline adherence.

The observed variation in performance across clinical specialties reflects the differential training and clinical focus of each discipline. General surgeons demonstrated the highest accuracy in treatment decisions (88.0%), consistent with their primary role in surgical management of acute cholecystitis. Gastroenterologists excelled in diagnostic questions (82.0%), reflecting their expertise in hepatobiliary pathophysiology and differential diagnosis. Emergency medicine physicians showed comparable overall performance despite having the broadest scope of practice, suggesting effective application of clinical decision-making frameworks in acute presentations. These findings are consistent with Gaber et al., who demonstrated that LLM workflows in clinical decision support vary in effectiveness across different clinical domains and specialties [5,6]. A methodological consideration is the inherent asymmetry between the NS-LLM system, which was explicitly optimized to follow TG18 through its symbolic knowledge base, and human participants, who were not provided with specific TG18 training prior to the study. This design reflects real-world clinical practice conditions but limits the interpretation of performance differences to guideline adherence rather than global clinical superiority. The observed performance gap may partly reflect differences in systematic guideline application rather than fundamental differences in clinical competence. It should be noted that the gold standard was established by a panel of three experts utilizing TG18 as the reference framework—the same guidelines encoded in the NS-LLM system’s symbolic knowledge base. This creates an inherent methodological alignment between the AI system and the evaluation criteria, potentially favoring the system’s performance. The observed accuracy differences should therefore be interpreted as reflecting superior guideline adherence in a structured assessment rather than comprehensive clinical superiority encompassing the full spectrum of physician competencies. Future studies comparing AI performance against physicians who have received immediate pre-study guideline refreshment would help disentangle guideline adherence from inherent diagnostic reasoning ability.

The clinical implications of our findings are significant, especially in resource-constrained environments where access to specialist expertise may be limited. Emerging evidence suggests that artificial intelligence tools can enhance clinical decision support, diagnostics, and workflow efficiency when incorporated into standard care procedures, thereby potentially improving patient outcomes and alleviating clinician workload in practical settings [18]. Our neuro-symbolic system’s consistent application of TG18 guidelines addresses the documented variability in guideline adherence (45–78%) across different healthcare settings [3]. The system’s ability to provide structured reasoning traces with explicit TG18 rule references enhances transparency and may facilitate clinician trust, which has been identified as a critical factor for AI adoption in healthcare [19]. Furthermore, the system’s performance on complex cases involving multi-organ dysfunction and special conditions demonstrates its potential utility in challenging clinical scenarios where cognitive load and time pressure may compromise human decision-making.

Importantly, the recent literature suggests that the optimal model for AI integration in clinical practice may involve augmentation rather than replacement of physician expertise. A randomized clinical trial found that the availability of a large language model to physicians as a diagnostic aid did not significantly improve their diagnostic reasoning compared with the use of conventional resources, while the large language model evaluated alone demonstrated higher performance than both physician groups [20]. This finding, known as “automation neglect” by some researchers, indicates that physicians may not effectively utilize AI recommendations even when available [21]. The structured output format of our neuro-symbolic system, which includes explicit confidence scores, clinical reasoning chains, and TG18 rule references, is designed to promote more effective human–AI collaboration by providing actionable and interpretable decision support.

The integration of the TG18 knowledge base as a structured symbolic component signifies a noteworthy progression beyond retrieval-augmented generation methodologies. Although retrieval-augmented LLMs have exhibited potential in clinical applications, a recent study assessing knowledge graph-enhanced LLMs for diagnostic support demonstrated enhanced clinician confidence and reasoning support, especially in cases that are rare or characterized by uncertainty. Our system extends this concept by encoding the complete TG18 diagnostic criteria, severity grading system, and management algorithms as interconnected nodes with explicit logical relationships, enabling rule-based inference that mirrors established diagnostic reasoning patterns. This approach ensures consistent guideline application across all queries while maintaining the flexibility to handle complex clinical scenarios involving multiple concurrent conditions.

Several limitations of this study warrant consideration. First, the evaluation was conducted using case-based multiple-choice questions, which may not fully capture the complexity of real-world clinical decision-making involving incomplete information, evolving presentations, and patient-specific factors. McCoy et al. demonstrated that LLMs show markedly lower performance on script concordance testing, which measures how new information adjusts diagnostic judgments under uncertainty, compared to standard medical multiple-choice benchmarks [22]. Second, the study population was limited to Turkish tertiary care centers, potentially limiting generalizability to other healthcare systems and cultural contexts. The exclusion of physicians with prior AI research experience, while intended to minimize performance bias, may have introduced selection bias by excluding a subpopulation of technologically engaged clinicians whose clinical reasoning patterns may differ from physicians without such experience. As AI tools become increasingly prevalent in clinical practice, the representativeness of study populations excluding AI-experienced physicians may diminish, and future studies should consider including such physicians as a separate comparison group. Third, the single-pass evaluation methodology does not capture the iterative nature of clinical reasoning, where diagnoses are refined based on response to treatment and additional investigations. Third, the sample size of 30 case-based questions, while generating 900 total human responses, may be insufficient to draw definitive conclusions and increases the risk of Type II error for subgroup analyses involving rare disease categories with limited question representation (Supplementary Table S1). Post hoc power analysis indicated that with 10 participants per group and an alpha level of 0.05, the study had approximately 80% power to detect a mean accuracy difference of 12 percentage points between groups (effect size d = 0.85), suggesting adequate power for the primary overall comparison but limited power for detecting smaller differences in subgroup analyses. Additional practical limitations warrant acknowledgment. First, the current system relies on structured, unambiguous clinical input and lacks the capability to process ambiguous or equivocal imaging reports that are frequently encountered in clinical practice, such as reports stating ‘findings suggestive of but not diagnostic for acute cholecystitis.’ The system’s semantic mapping module is designed for structured entity extraction rather than probabilistic interpretation of diagnostic uncertainty. Second, the weight allocation for patients’ comorbidities within the symbolic knowledge base follows predefined scoring systems (CCI and ASA-PS) and does not account for nuanced clinical interactions between multiple comorbid conditions that may influence surgical risk beyond standardized indices. For example, the synergistic risk of combined hepatic and renal dysfunction may exceed the additive risk captured by these scoring systems. Third, the system lacks a mechanism to integrate patient preferences, values, and goals of care into its treatment recommendations, which is a fundamental component of shared decision-making in contemporary medical practice and an ethical imperative in surgical planning. From a technical standpoint, the NS-LLM system requires access to cloud-based API endpoints for both constituent large language models (Gemini 3.0 Flash via Google Cloud and GPT-5.2 via OpenAI API), necessitating stable internet connectivity and incurring per-query computational costs. The LangGraph orchestration framework requires Python 3.10 or higher with compatible dependency libraries. While the system does not require local high-performance GPU infrastructure (as neural inference is handled by cloud-based APIs), this reliance on third-party cloud services introduces dependencies on service availability, network latency, and potential API deprecation or pricing changes. The symbolic knowledge base traversal and Monte Carlo dropout-based uncertainty quantification impose additional computational overhead that may limit real-time applicability in high-throughput clinical settings. These infrastructure dependencies may restrict deployment in resource-limited healthcare environments with inadequate computational infrastructure or unreliable network connectivity. Finally, the neuro-symbolic system’s performance is inherently constrained by the completeness and currency of its symbolic knowledge base, requiring periodic updates to incorporate evolving clinical guidelines.

Future research should focus on prospective validation of the neuro-symbolic system in real-world clinical settings, evaluation of its impact on patient outcomes, and assessment of clinician acceptance and workflow integration. The development of multi-agent collaborative systems incorporating diverse clinical perspectives, as proposed by the AI Agent Hospital concept, represents a promising direction for comprehensive clinical decision support [23]. Current research in medical artificial intelligence is increasingly directed toward multimodal fusion integrating clinical text, imaging, and genomic data, enhancement of clinical interpretability through explainable AI methodologies, and improvement of cross-scenario generalization capabilities that enable robust performance across diverse clinical settings and patient populations [24,25]. These research directions align with the neuro-symbolic approach employed in the present study, which enhances interpretability through explicit symbolic reasoning while maintaining the pattern recognition capabilities of neural networks. Future iterations of the NS-LLM system may benefit from incorporating multimodal input processing to simultaneously analyze clinical text, laboratory data, and imaging findings within a unified reasoning. In the current study design, clinical vignettes were presented as structured text input, requiring manual curation of clinical scenarios. Real-world clinical deployment of the NS-LLM system would necessitate the development of interoperability pipelines compatible with hospital electronic health record (EHR) systems (e.g., HL7 FHIR-based data exchange) and integration with imaging report systems (PACS/RIS) for automated extraction of radiology findings. The requirement for manual data preprocessing represents a significant barrier to seamless clinical workflow integration. While the present study utilized hypothetical clinical vignettes without real patient data, future clinical deployment of the NS-LLM system would necessitate comprehensive data protection measures. These should include end-to-end data encryption (e.g., AES-256) for data in transit and at rest, compliance with international privacy regulations including the Health Insurance Portability and Accountability Act (HIPAA) and the General Data Protection Regulation (GDPR), implementation of de-identification and pseudonymization protocols for clinical data processing, role-based access control mechanisms, comprehensive audit logging of all system interactions with patient data, and institutional data governance frameworks with regular security assessments. The current prototype operates through cloud-based API endpoints (Google Cloud and OpenAI API), which introduces additional data sovereignty and third-party processing considerations that must be addressed through Business Associate Agreements (BAAs) and Data Processing Agreements (DPAs) in compliance with applicable regulations. The absence of these safeguards in the current prototype represents a limitation that must be addressed prior to any clinical implementation

## 5. Conclusions

In conclusion, this multi-center study demonstrates that a neuro-symbolic LLM system incorporating TG18 guidelines achieves high diagnostic accuracy in standardized case-based assessments of acute cholecystitis management. However, these findings should be interpreted within the context of a structured multiple-choice evaluation, which does not fully capture the complexity of real-world clinical decision-making involving patient preferences, evolving clinical presentations, incomplete information, and the multidimensional physician-patient relationship. The NS-LLM system’s performance advantage primarily reflects its systematic and consistent application of encoded guidelines, rather than a demonstration of global clinical superiority over human experts. Artificial intelligence systems, including neuro-symbolic architectures, should be regarded as decision-support tools that augment rather than supplant physician expertise. The ultimate clinical responsibility for patient care—encompassing diagnostic reasoning, therapeutic decision-making, empathic communication, and the integration of patient values into management plans—remains firmly within the domain of the human clinician. The consistent application of standardized guidelines by the NS-LLM system may help reduce the documented variability in acute cholecystitis management across healthcare settings, particularly in resource-limited environments where specialist expertise is unavailable.

## Figures and Tables

**Table 1:** Demographic and Professional Characteristics of Human Participants.

Characteristic	General Surgery (n = 10)	Gastroenterology (n = 10)	Emergency Medicine (n = 10)	*p*-Value
Age, years, mean ± SD	42.3 ± 5.8	44.1 ± 6.2	40.8 ± 5.4	0.412
Male sex, n (%)	7 (70)	6 (60)	6 (60)	0.872
Clinical experience, years, mean ± SD	11.2 ± 4.3	12.8 ± 5.1	10.5 ± 3.8	0.528
**Academic position, n (%)**				**0.634**
Assistant Professor	3 (30)	4 (40)	2 (20)	
Associate Professor	2 (20)	2 (20)	1 (10)	
Specialist Physician	5 (50)	4 (40)	7 (70)	
**Institution, n (%)**				**1.000**
Ankara Bilkent City Hospital	3 (30)	3 (30)	3 (30)	
Ankara Etlik City Hospital	3 (30)	3 (30)	3 (30)	
Elazığ Fethi Sekin City Hospital	2 (20)	2 (20)	2 (20)	
Etimesgut State Hospital	2 (20)	2 (20)	2 (20)	
Annual cholecystectomies, mean ± SD †	124 ± 45	N/A	N/A	—
Annual acute biliary cases, mean ± SD	156 ± 52	98 ± 38	215 ± 78	**0.023**

SD, standard deviation; N/A, not applicable. † Performed as primary surgeon. *p*-values calculated using one-way ANOVA for continuous variables and chi-square test (or Fisher’s exact test where expected cell frequencies < 5) for categorical variables. Bold *p*-value indicates statistical significance (*p* < 0.05).

**Table 2:** Overall Diagnostic Accuracy by Participant Group.

Group	Correct Responses	Accuracy, %	95% CI	AUC	*p*-Value *
**NS-LLM System**	**29/30**	**96.7**	**82.8–99.9**	**0.983**	**Reference**
General Surgery (n = 10)	24.7 ± 2.0	82.3 ± 6.8	75.5–89.1	0.856	<0.001
Gastroenterology (n = 10)	23.6 ± 2.2	78.7 ± 7.4	71.3–86.1	0.821	<0.001
Emergency Medicine (n = 10)	21.3 ± 2.5	71.0 ± 8.2	62.8–79.2	0.764	<0.001

CI, confidence interval; AUC, area under the receiver operating characteristic curve. Values for human groups are expressed as mean ± SD. * *p*-values for comparison with NS-LLM system using independent samples *t*-test with Bonferroni correction. Bold row indicates the reference system (NS-LLM).

**Table 3:** Diagnostic Accuracy by Clinical Domain.

Clinical Domain	NS-LLM System	General Surgery (n = 10)	Gastroenterology (n = 10)	Emergency Medicine (n = 10)
***Diagnosis (n = 10 questions)***				
Accuracy, %	**100.0**	78.0 ± 8.2	82.0 ± 7.5	74.0 ± 9.1
Correct responses	10/10	7.8 ± 0.82	8.2 ± 0.75	7.4 ± 0.91
***Treatment (n = 10 questions)***				
Accuracy, %	**90.0**	88.0 ± 6.3	76.0 ± 8.4	70.0 ± 7.8
Correct responses	9/10	8.8 ± 0.63	7.6 ± 0.84	7.0 ± 0.78
***Complications/Prognosis (n = 10)***				
Accuracy, %	**100.0**	81.0 ± 7.1	78.0 ± 6.8	69.0 ± 8.5
Correct responses	10/10	8.1 ± 0.71	7.8 ± 0.68	6.9 ± 0.85

Values for human groups are expressed as mean ± SD. The NS-LLM system maintained significant superiority over all human groups in each domain (all *p* < 0.01). Bold indicates the highest accuracy value in each domain.

**Table 4:** Diagnostic Accuracy by Disease Category.

Disease Category	n	NS-LLM	Surgery	Gastro	Emergency
Acute Calculous Cholecystitis	6	**100.0**	85.0 ± 5.8	80.0 ± 6.3	73.3 ± 8.2
Acute Acalculous Cholecystitis	2	**100.0**	80.0 ± 10.5	85.0 ± 7.1	70.0 ± 12.2
Complicated Cholecystitis †	5	**100.0**	88.0 ± 5.4	82.0 ± 6.9	72.0 ± 9.3
Chronic Cholecystitis/Biliary Colic	4	**100.0**	85.0 ± 7.1	77.5 ± 8.5	75.0 ± 6.5
GB Polyps/Adenomyomatosis	3	**100.0**	76.7 ± 11.5	80.0 ± 8.2	66.7 ± 12.5
Mirizzi Syndrome	2	**100.0**	80.0 ± 14.1	75.0 ± 7.1	65.0 ± 14.1
Gallbladder Carcinoma	4	87.5	80.0 ± 8.2	77.5 ± 9.6	70.0 ± 8.2
Post-Cholecystectomy Complications	4	87.5	82.5 ± 6.5	75.0 ± 7.1	72.5 ± 9.6

GB, gallbladder; Gastro, gastroenterology. † Includes gangrenous, emphysematous, and perforated variants. Values are expressed as accuracy percentage (mean ± SD for human groups). Bold indicates 100% accuracy achieved by the NS-LLM system.

**Table 5:** NS-LLM System Confidence Scores and Inter-Agent Agreement.

Metric	Overall (n = 30)	Correct (n = 29)	Incorrect (n = 1)	*p*-Value
***Mean Confidence Score***				
Overall	0.924 ± 0.048	0.931 ± 0.042	0.724	0.031
Agent 1 (Gemini 3.0 Flash)	0.918 ± 0.052	0.926 ± 0.046	0.682	0.028
Agent 2 (GPT-5.2)	0.931 ± 0.045	0.938 ± 0.039	0.766	0.042
***Inter-Agent Agreement***				
Full agreement, n (%)	27 (90.0)	27 (93.1)	0 (0)	<0.001
Partial agreement, n (%)	3 (10.0)	2 (6.9)	1 (100)	
***Reproducibility***				
Cohen’s kappa (inter-run)	0.967	—	—	—
Identical answers across runs, n (%)	29 (96.7)	—	—	—

Confidence scores range from 0 to 1. Values are expressed as mean ± SD unless otherwise indicated. Inter-run agreement assessed across three independent system evaluations with temperature parameter set to 0.1. — indicates not applicable.

## Data Availability

The raw data supporting the conclusions of this article will be made available by the authors on request.

## Supplementary Materials

The following supporting information can be downloaded at: https://www.mdpi.com/article/10.3390/jcm15051730/s1, Table S1: Complete dataset containing all 30 clinical vignettes with question domains, difficulty levels, correct answers, TG18-based explanations, NS-LLM system responses with dual-agent confidence scores and agreement status, and individual responses, correctness, and response times for all 30 human participants (10 general surgeons, 10 gastroenterologists, 10 emergency medicine physicians); Table S2: Question-level analysis summarizing per-question accuracy rates and mean response times stratified by clinical specialty, with overall human error rates; Table S3: Individual participant performance statistics including total and domain-specific accuracy (diagnosis, treatment, complications), mean response times, and range for each of the 30 clinicians; Table S4: Overall performance summary presenting group-level accuracy, standard deviations, and 95% confidence intervals for the NS-LLM system and each physician specialty, with pairwise statistical comparisons and subgroup analyses by clinical domain and difficulty level; Table S5: NS-LLM system reproducibility data from all three independent runs, detailing per-question responses, correctness, calibrated confidence scores, individual agent outputs (Agent 1: Gemini 3.0 Flash; Agent 2: GPT-5.2), inter-agent agreement status, and processing times.
